# Artificial intelligence in patient-specific hand surgery: a scoping review of literature

**DOI:** 10.1007/s11548-023-02831-3

**Published:** 2023-01-12

**Authors:** Marco Keller, Alissa Guebeli, Florian Thieringer, Philipp Honigmann

**Affiliations:** 1 Hand Surgery, Department of Orthopaedic Surgery and Traumatology, Kantonsspital Baselland, 4410 Liestal, Switzerland; 2 Medical Additive Manufacturing Research Group, Department of Biomedical Engineering, University of Basel, 4123 Allschwil, Switzerland; 3 Department of Plastic and Hand Surgery, Kantonsspital Aarau, 5001 Aarau, Switzerland; 4 Department of Oral and Cranio-Maxillofacial Surgery, University Hospital Basel, Basel, Switzerland; 5 Department of Biomedical Engineering and Physics, Amsterdam UMC, University of Amsterdam, Amsterdam, The Netherlands

**Keywords:** Hand surgery, Hand rehabilitation, Artificial Intelligence, Machine learning, Deep learning, Mathematical modelling

## Abstract

**Purpose:**

The implementation of artificial intelligence in hand surgery and rehabilitation is gaining popularity. The purpose of this scoping review was to give an overview of implementations of artificial intelligence in hand surgery and rehabilitation and their current significance in clinical practice.

**Methods:**

A systematic literature search of the MEDLINE/PubMed and Cochrane Collaboration libraries was conducted. The review was conducted according to the framework outlined by the Preferred Reporting Items for Systematic Reviews and Meta-Analysis Extension for Scoping Reviews. A narrative summary of the papers is presented to give an orienting overview of this rapidly evolving topic.

**Results:**

Primary search yielded 435 articles. After application of the inclusion/exclusion criteria and addition of supplementary search, 235 articles were included in the final review. In order to facilitate navigation through this heterogenous field, the articles were clustered into four groups of thematically related publications. The most common applications of artificial intelligence in hand surgery and rehabilitation target automated image analysis of anatomic structures, fracture detection and localization and automated screening for other hand and wrist pathologies such as carpal tunnel syndrome, rheumatoid arthritis or osteoporosis. Compared to other medical subspecialties the number of applications in hand surgery is still small.

**Conclusion:**

Although various promising applications of artificial intelligence in hand surgery and rehabilitation show strong performances, their implementation mostly takes place within the context of experimental studies. Therefore, their use in daily clinical routine is still limited.

## Introduction

There is a rapidly growing number of applications of artificial intelligence (AI) in the medical field. To comprehend the fundamentals of the use of AI for different purposes, one must deal with some basic terminology: The term “Artificial Intelligence” was coined by Arthur McCarthy in 1955 [1]. Nowadays it can be understood as an umbrella term for the application of algorithms that provide machines with the ability to solve problems that traditionally required human intelligence [2]. AI’s abilities are based on algorithms performing pattern recognition and self-correction using vast amounts of data.

Some frequently encountered subfields of AI are “machine learning” (ML), which describes the ability of algorithms to learn from data, or “deep learning” (DL), which is a subfield of ML and stands for the use of multiple layers of artificial neural networks. ML happens by optimization of a mathematical model. These models are trained to yield optimal predictions on a so called training dataset. The training can either happen supervised (pre-labelled data by humans set as the “ground truth” and used by the algorithm to learn) or unsupervised (the input data are not labelled and the algorithm autonomously seeks ways to find meaningful structure in the data). DL uses artificial neural networks in which multiple network layers are added to increase the levels of abstraction and performance, so-called convolutional neural networks (CNN). Currently, DL is the state-of-the-art for unsupervised models on image data [3, 4].

**Fig. 1:**
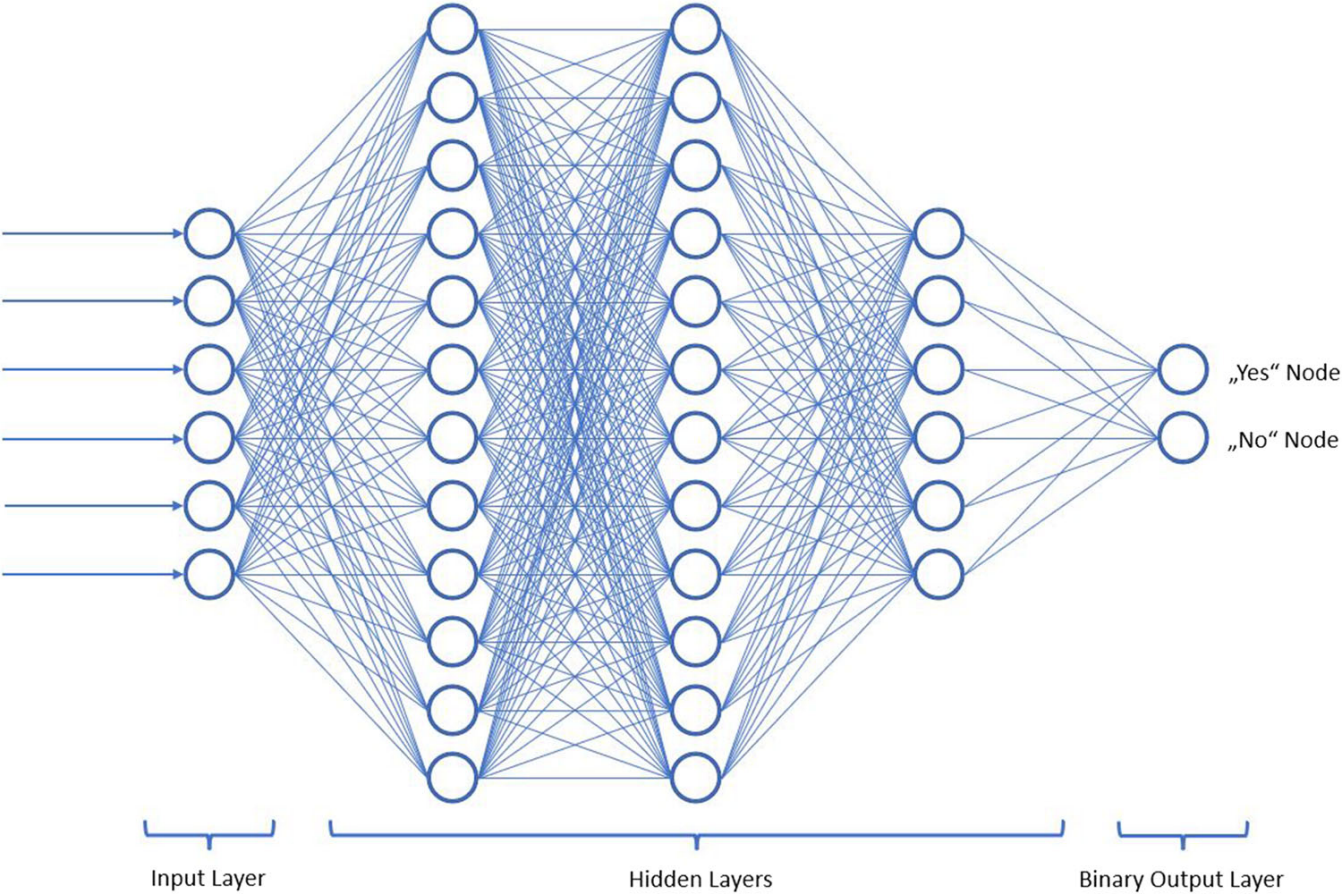
Simplified schematic pattern of an artificial neural network (ANN)

Studies assessing AI-based algorithms’ ability of performing certain tasks usually contain large amount of input data which are split into a “training set”, a “validation set” and a “test set”. A common ratio of splitting the data is 80:10:10. After a training phase of several cycles (“epochs”), the algorithm is exposed to the test set. The performance is then assessed using parameters like “the area under the receiver operator curve” (AUC, where an AUC of 1.0 would indicate perfect prediction and 0.5 would indicate a prediction equivalent to the flipping of a coin), sensitivity and specificity. In order to compare the algorithm’s performance to human performance, a designated group of test subjects is exposed to the same test set. Nowadays, convolutional neural networks are highly complex and in retrospect, it is usually not intuitively comprehensible how a suggestion was made. A simplified illustration of a fully-connected neural network can be divided into an input layer, a series of interconnected hidden layers (“neurons”) which collect, organize and process the data before passing it on to the next layer. This continues layer by layer and culminates in an output layer, based on which a suggestion or prediction is formulated (Fig. 1).

Building and training effective CNNs from scratch requires a huge amount of data and is usually not feasible for the majority of clinical researchers. To overcome this hurdle, it is possible to adopt features from powerful pre-trained CNNs (e.g. open source CNNs) and train them for a new task (“transfer-learning”) [3].

Common applications of artificial intelligence in general orthopaedics are image recognition (fracture detection/classification), preoperative risk assessment, clinical decision-making or evaluating probabilities of certain outcomes after treatment [4, 5] A systematic review of 12 studies on fracture detection using AI in general orthopaedics highlighted a promising performance with near perfect prediction in five articles (AUC 0.95–1.0) [5].

The rationale of this scoping review was to investigate the current extent of the implementation of artificial intelligence in the field of hand surgery and rehabilitation. The objectives of this review were to create an overview of the topic and then evaluate the current significance of artificial intelligence in hand surgery and rehabilitation daily hospital routine.

## Materials and methods

### Search strategy

To conduct this review, the checklist suggested by the Preferred Reporting Items for Systematic Reviews and Meta-Analysis Extension for Scoping Reviews (PRISMA-ScR) was utilized [6]. By following the suggested framework for scoping reviews, we intended to give an informative overview and allow the reader to find a systematic approach to this very heterogenous topic.

First we conducted a systematic search of the MEDLINE/PubMed and Cochrane Collaboration libraries. For the primary search, we used the following keywords in the title: (automat* OR computer-aid* OR “artificial intelligence” OR “neural network” OR “machine learning” OR “deep learning”) AND (“hand surgery” OR hand OR wrist OR finger). Given the fact of often inconsistent terminology in the literature about artificial intelligence we decided to use six commonly used terms combined with four hand surgery related terms with the hope to identify the vast majority of the relevant articles with the primary search using the combination of those terms. The search was conducted on the 04.07.2021. The titles were screened by two independent reviewers. The inclusion and exclusion criteria, which are highlighted in Table 1, were then applied (Table 1). We excluded review articles, letters to the editor, conference abstracts and articles not published in English. Disagreement concerning the inclusion of articles was resolved by consensus between the two investigators. In addition, all included studies underwent supplemental manual bibliographic review to identify additional studies that may be relevant and were missed initially.

**Table 1:** Inclusion and exclusion criteria of the systematic review

Inclusion criteria	Exclusion criteria
Articles on the implementation of automated image analysis in hand surgery and rehabilitation	Articles not related to hand surgery and rehabilitation
Articles on the implementation of artificial intelligence in hand surgery and rehabilitation	Review articles
	Letters to the editor
	Conference abstracts
	Language other than English

### Quality assessment

The focus of this systematic search was to create an overview of all the different applications of the above-mentioned technologies in hand surgery-related areas. The articles met the inclusion criteria if they addressed the implementation of automation (mathematical modelling) or artificial intelligence in any hand surgery and rehabilitation-related area. Therefore, in the title review all articles not hand surgery and rehabilitation-related at all (e.g. “hand-and-foot disease” or “hand hygiene”) were excluded. We did not restrict the search to automated fracture detection or fracture classification.

## Results

The initial search generated 435 articles of which 212 remained after title review and application of the inclusion and exclusion criteria. The supplemental manual search yielded 23 more articles. Finally, 235 articles were eligible for this review (Table 2).

**Table 2:**
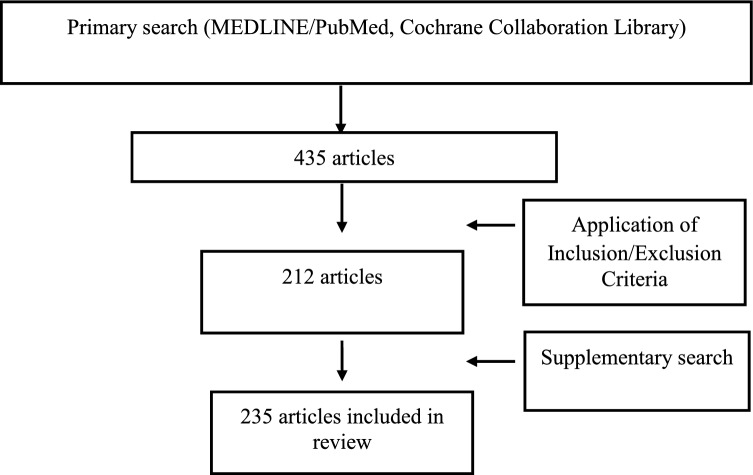
Flow chart for database research

Since full text review of 235 articles was not feasible, the titles and abstracts of the remaining articles were screened. At this point, we recognized the broad spectrum of the articles’ topics and the difficulty of defining which ones are actually hand surgery and rehabiliation-related and which are not. To achieve the goal of this review, we decided to build subgroups and elucidate the articles on topics which, in our opinion, could be relevant for the daily routine of a hand surgeon, in detail, and mention the articles rather loosely related to hand surgery in a summarizing matter.

### Automated image analysis (using mathematical modelling) of anatomic structures

**Fig. 2:**
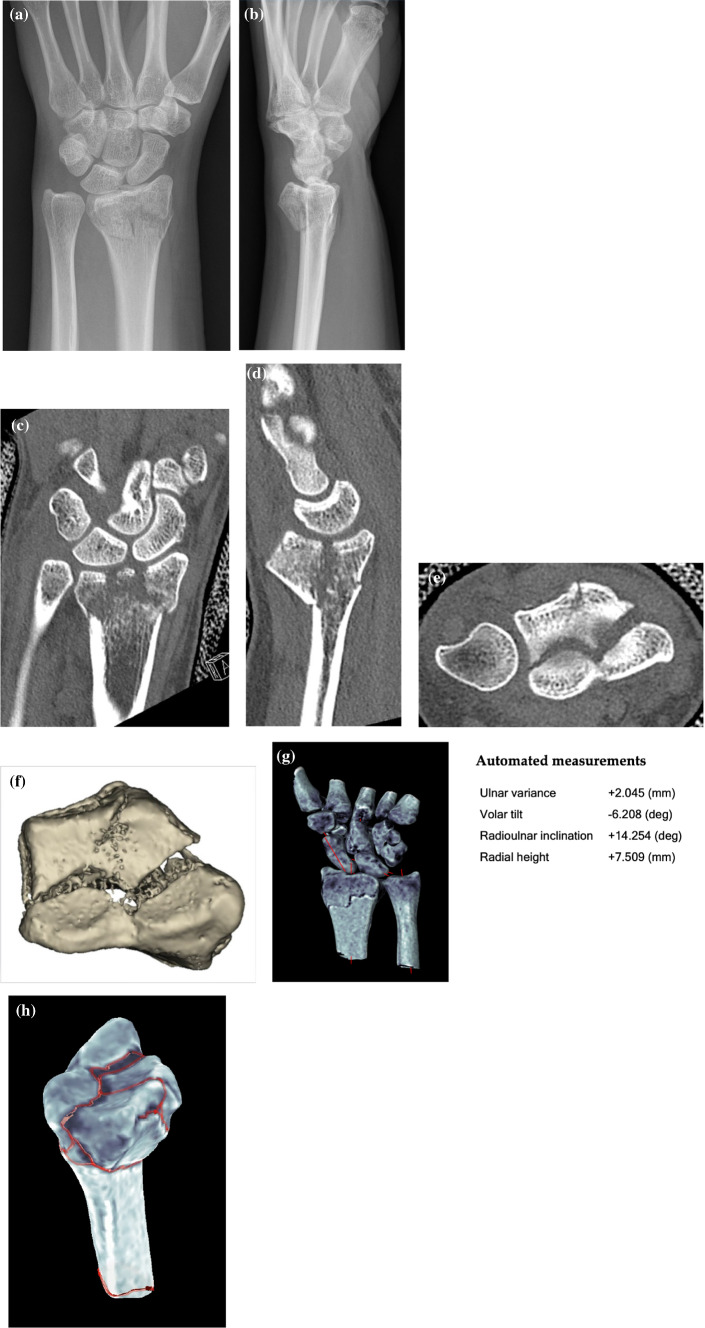
**a, b** Dorsopalmar and lateral projection of radiographs of a 43-year-old female who sustained an intraarticular distal radius fracture by falling on her extended left (adominant) wrist. **c–e** Coronar, sagittal and axial view of computed tomography images of the wrist of the same patient showing the fracture dislocation and multiple intraarticular fragments. **f** Three-dimensional reconstruction of the computed tomography images showing the intra-articular fragments and gaps in the articular surface. **g** Automated measurements conducted by the Bonelogic® 2 software (Disior Ltd, Helsinki, Finland). **h** Screenshot of the same software conducting automated fracture line detection

**Fig. 3:**
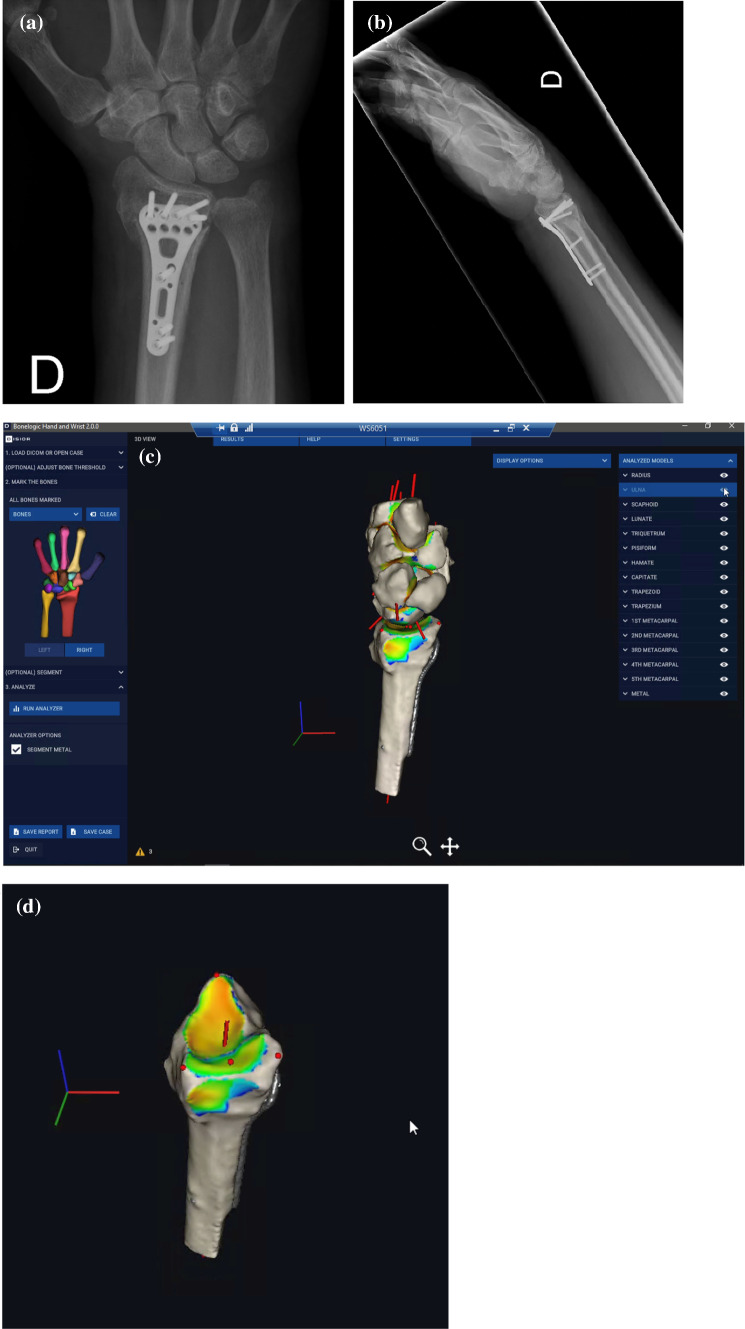
**a, b** Dorsopalmar and lateral projection of radiographs of a 72-year old male with malunion of a distal radius fracture after open reduction and internal fixation with a volar plate. **c** Automated three-dimensional assessment of the distal radius malunion with distance mapping of radiocarpal and intercarpal relationships. **d** Automated three-dimensional assessment of the distal radius malunion with colour mapping showing increased load on the dorsal aspect of the articular surface

In 2020 the research group around Nora Suojärvi and Eero Waris et al. presented a newly developed wrist application of an image processing software, which was already available for foot and ankle analysis (Bonelogic® 2, Disior Ltd, Helsinki, Finland). The program uses *mathematical modelling to record the 3D geometry of wrist and carpal bones*, based on cone-beam computed tomography (CBCT) images: after acquisition of a CBCT scan of a patient’s wrist, the bones have to be labelled manually. The software then automatically identifies the location of measurement landmarks and determines the longitudinal axis for each bone. This is achieved through analysis of the bones’ cross section at various locations. The software then applies robust line fitting routines to convert the centre curve into a straight-line representative [7]. Local shape information of the radius model is used to identify certain surface points, with which frequently used radiological parameters of the distal radius such as radial inclination, radial height, dorso-palmar tilt and ulnar variance are determined. This can be executed on healthy radii or even in the presence of a fracture, to assess the extent of dislocation. The reliability of the software was examined with the analysis of 50 uninjured radii and compared with interobserver agreement and intra-rater reliability of measurements conducted by three groups of physicians carried out on plain radiographs. The reliability of the algorithm was found to be excellent, whereas the physicians showed substantial inter-observer variability in interpreting the angular parameters of the healthy radii [8, 9]. The newest version of the software is also able to automatically measure intercarpal relationships such as scapho-lunar or luno-triquetral distance and scapho-lunar angle. Two examples of the application of automated image analysis in clinical cases are shown in Figs. 2 and 3.

Roner et al. developed a similar *three-dimensional automated assessment of the distal radioulnar joint (DRUJ) morphology* which was used to measure ulnar variance and anatomical characteristics of the sigmoid notch. The method was used on 53 healthy forearms and automated measurements were compared along three different imaging modalities (plain radiographs, computed tomography scans, 3-dimensional printed models). The authors found the method to be valuable in surgical planning of procedures involving the DRUJ, e.g. length correction of the ulna or ulna head arthroplasty. Furthermore, they stated that their developed method could be more observer-independent than current standard methods. One of the limitations was that the estimated cartilage area could not be assessed precisely since the method relies on scans of osseous structures. Another limitation was that the landmarks to calculate DRUJ measurements had to be placed manually by an expert orthopaedic surgeon, which highlights the need for automated region and landmark identification [10].

### Fracture detection using machine learning

Similar to general orthopaedics, the most common application of artificial intelligence in hand surgery targets fracture detection.

The literature search yielded two articles on *automated detection of scaphoid fractures*. Despite thorough clinical examination and multiple radiographic projections, a substantial number of scaphoid fractures (16%) are missed on initial plain radiographs due to the lack of clearly visible fracture lines [11]. This emphasizes the need for assistance, such as artificial intelligence, in order to improve the diagnostic accuracy.

In 2019, Langerhuizen et al. compared the performance of a deep learning algorithm (convolutional neural network) to the performance of five orthopaedic surgeons in detecting scaphoid fractures using sets of four radiographic imaging views. Three hundred radiographic scaphoid series were retrospectively identified, including 150 fractures (127 visible on radiographs and 23 only visible on MRI) and 150 non-fractures. All image series had corresponding CT or MRI to confirm the presence or absence of a fracture. An open source pretrained CNN (Visual Geometry Group, Oxford, United Kingdom) was used [12]. The algorithm showed an AUC of 0.77, 72% accuracy, 84% sensitivity and 60% specificity. The performance could not be improved by adding demographic data (age and sex). The human observers showed a better specificity (93%), while accuracy (84%) and sensitivity (76%) were similar to the ones of the algorithm. Although the CNN was able to detect five of six occult scaphoid fractures that were missed by all orthopaedic surgeons, it also made thirteen false positive suggestions, which were all correctly detected by the human observers. The authors concluded that the algorithm had difficulties identifying scaphoid fractures that are obvious to human observers. Some limitations of this study are the small dataset size and, since only image series with corresponding CT or MRI pictures were eligible, a selection bias (mostly hard to detect or occult scaphoid fractures). To improve the specificity, they suggested further refinement of the algorithm by adding information from physical examination or training of the CNN with a larger dataset [5].

In 2020, another article on a similar topic was published by Ozkaya et al. The aim was to evaluate the diagnostic performance of a CNN for detecting scaphoid fractures on anteroposterior wrist radiographs. The performance of the CNN was compared to the performance of an emergency department physician and two orthopaedic specialists (one experienced in hand surgery and one less experienced). Three hundred and ninety anteroposterior (AP) wrist radiographs (192 scaphoid fractures and 198 normal wrists) were included into the dataset. The CNN showed 76% sensitivity, 92% specificity and 0,840 AUC, which was considered “acceptable” by the authors. The algorithm especially had difficulties in detecting occult scaphoid fractures. The orthopaedic surgeon experienced in hand surgery demonstrated the best diagnostic performance, whereas the CNN’s performance was similar to the less experienced orthopaedic surgeon. The emergency department physician showed the worst diagnostic performance. Therefore, the authors highlighted the potential use of artificial intelligence in detecting scaphoid fractures, especially in the absence of an experienced orthopaedic or hand surgeon [13].

Apart from scaphoid fractures, there’s a group of articles on the use of *convolutional neural networks for the detection of distal radius fractures*: Gan et al. investigated the use of a fast object detection algorithm based on deep learning to identify the distal radius on anteroposterior wrist radiographs as the region of interest and to detect a possible distal radius fracture. The dataset contained 2340 wrist radiographs and the diagnostic performance was compared to that of orthopaedic surgeons and radiologists. The algorithm had a 100% success rate in automatically detecting the region of interest. This aspect makes this algorithm stand out from most other algorithms, since the images in datasets of other studies mostly have to be cropped and resized manually before they can be processed by a CNN. In distinguishing normal radiographs from distal radius fractures, it showed an accuracy of 93%, sensitivity of 90% and specificity of 96%. Its diagnostic performance was similar to the one of the orthopaedic surgeons and superior to the one of the radiologists. After reviewing some of the radiographs, which were wrongfully labelled as “normal” by the CNN, the authors noted that on the anteroposterior radiograph there were no apparent fracture signs (displaced fragments or fracture lines), but they would have been visible on the corresponding lateral views. They concluded that the addition of the lateral radiograph, which depicts a more realistic diagnostic workflow, would have improved the CNN’s performance [14].

The strong performance of CNNs in detecting distal radius fractures was also highlighted by a number of further studies: Kim and MacKinnon presented a CNN trained to detect distal radius fractures with a dataset of 1389 lateral wrist radiographs. The AUC was 0.954, sensitivity and specificity were 0.9 and 0.88, respectively. Again, the authors concluded that the addition of a second projection (anteroposterior view) would have improved the algorithms performance [3]. Thian et al. presented an object detection CNN for fracture detection which was trained on 7356 wrist radiographs. Radius and ulna fractures were first annotated with bounding boxes by radiologists. The model detected and correctly localized 91.2% and 96.3% of all radius and ulna fractures on the dorsopalmar and lateral projection, respectively [15].

Besides the mentioned articles, which are exclusively about fracture detection in the wrist, our search yielded a number of studies which, beside other body parts, also contained radiographs of hands or wrists. These articles are not exclusively on hand surgery but nevertheless contain valuable information for hand surgeons: Olczak et al. exposed five freely available deep learning algorithms to 256′458 radiographs (hand, wrist and ankle) with associated radiologist reports, with 56% of the images containing fractures. The algorithm was trained in fracture detection. Again, only one radiological projection was used. The best performing network achieved an accuracy of 83%, which was comparable to the performance of two senior orthopaedic surgeons [16].

Robert Lindsey et al. published some articles on their experiments with convolutional neural networks trained on datasets of 135′845 radiographic images of different bodyparts. Besides fracture detection, the CNN was also trained to localize the fracture and highlight it with a heatmap. The authors were able to demonstrate that the accuracy in fracture detection by emergency medicine clinicians could be significantly improved with the aid of mentioned CNN [17]. The group also presented their results with a subset of 36′408 wrist radiographs. The network had an AUC of 0.97, a specificity of 93% and sensitivity of 95%. The algorithm’s diagnostic performance was significantly better than the one of trainees, physician extenders or practicing urgent care physicians, suggesting that the proposed technology would be especially helpful in residency training and urgent care clinical settings [17, 18].

Table 3 gives an overview over publications on automated fracture detection in the hand and wrist and the reported performance metrics.

**Table 3:** A comprehensive (to our knowledge) compilation of published research projects targeting automated fracture detection in the hand or wrist using machine learning approaches (*AUC* area under the ROC (receiver operator characteristic) curve; *dp* dorsopalmar radiograph projection; *lat* lateral radiograph projection)

Study	Task (fracture detection)	Dataset size (radiographs, views)	AUC	Sensitivity	Specificity
Olczak [16]	Any fracture	256′000 (Wrist, Hand and Ankle radiographs, all views)	0.83	–	–
Kim [3]	Distal radius or distal ulna fracture	1389 (wrist, lat)	0.954	0.9	0.88
Lindsey [17]	Any fracture	34,990 (wrist, dp and lat)	0.990	0.939	0.945
Gan [14]	Distal radius fracture	2340 (wrist, dp)	0.93	0.9	0.96
Thian [15]	Distal radius or ulna fracture	7356 (wrist, dp and lat)	0.918 (dp) 0.933 (lat)	0.957 (dp). 0.967 (lat)	0.825 (dp) 0.864 (lat)
Blüthgen et al. [19]	Distal radius fracture	524 (wrist, dp and lat)	Internal test set: 0.93–0.96 External test set: 0.80–0.89	Internal test set: 0.81–0.90 External test set: 0.64–0.92	Internal test set: 0.86–1.0 External test set: 0.60–0.90
Langerhuizen [5]	Scaphoid fracture	300 (wrist, 4 views (= scaphoid series))	0.77	0.84	0.60
Ozkaya [13]	Scaphoid fracture	390 (wrist, dp)	0.84	0.76	0.92
Hendrix [20]	Scaphoid fracture	4229 (hand, wrist, scaphoid)	0.87	0.78	0.84

### Other applications of artificial intelligence in hand surgery

Apart from fracture detection, there are some other remarkable applications of AI in hand surgery. This is just a selection of the identified articles:

Tecle et al. presented the use of a *CNN to diagnose osteoporosis based on cortical thickness of the second metacarpal bone* on anteroposterior radiographs of the hand. Furthermore, the presented algorithm was able to autonomically correct the laterality and vertical alignment of the radiographs for better image processing [21]. Kuok et al. developed a *computer-aided soft tissue segmentation method using a deep convolutional neural network*, to help distinguish tendon tissue from synovial sheath tissue in ultrasound images. The authors suggested the potential use of the method in diagnosis and ultrasound-guide treatment of trigger finger [22].

Saun et al. showed that CNNs can also be used to *automatically classify hand radiographs according to their positioning*. The suggested algorithm was able to distinguish between anteroposterior, oblique and lateral hand radiographs with an accuracy of 96.0% [23].

Papez et al. published an article on *infrared thermography based on artificial intelligence for diagnosis of Carpal Tunnel Syndrome diagnosis*. The authors concluded that the method cannot be recommended as an adequate diagnostic tool for exact severity quantification, but could be useful as a screening tool for severe cases [24].

### Applications of artificial intelligence loosely related to hand surgery

The systematic search yielded numerous articles about *computer-aided goniometry and hand movement analysis*: 91 articles covered topics such as automated motion pattern detection or hand gesture classification. The movements or hand poses were captured with all kinds of input devices (photographs, video analysis, infrared images, surface vibrations, surface EMG measurement, wearable data gloves and other kind of body-mounted sensors). In most cases the evaluation of the data was conducted using machine learning. One example of a practical application is the quantification of tremor with deep learning for neurological disease diagnosis [25]. Another example is the article published by Anaz et al. [26]: In order to facilitate automated active range of motion (AROM) measurements, the authors presented an algorithm using convolutional neural networks to detect hand poses. The CNN showed 99% accuracy in detecting the correct of eight possible hand poses and required an average of 8 ms for this task [25]. There is a substantial overlap of these kinds of articles with research on myoelectric prostheses, which are controlled by computer-aided motion analysis [27, 28].

The second largest group of articles is on *the computer-aided diagnosis and screening of rheumatoid arthritis*. 30 articles were found on this topic. These studies mostly suggest a method of detecting rheumatoid arthritis by automated analysis of joint space narrowing on hand radiographs using artificial neural networks. For example, Üreten et al. presented an artificial neural network with an accuracy of 73.33%, sensitivity of 0.6818 and specificity of 0.7826. Based upon these performance metrics, the authors concluded that the use of convolutional networks in diagnosing rheumatoid arthritis is promising [29]. Some other similar algorithms use automated analysis of synovitis in ultrasound images or magnetic resonance imaging for the diagnosis or activity scoring of rheumatoid arthritis.

16 studies presented similar methods of *automated bone age assessment from hand radiographs* using neural networks. These CNNs are usually trained on large datasets and show very good performances with a discrepancy of only a couple of months compared to manual bone age assessment [30]. For example, Spamimpato et al. presented an algorithm with an average discrepancy between manual and automated age assessment of 0.8 years, which was considered to be a state-of-the-art performance [31].

## Discussion

Artificial intelligence in healthcare is rapidly expanding. The number of related articles has grown exponentially throughout the last 20 years. Yet, there is debate about the significance of AI in clinical daily routine. A systematic review on the role of AI in real-life clinical practice found that in respect of the wide range of clinical tasks on which AI can be applied, most of the published articles are proof-of-concept studies which focus on the development of an algorithm and validating it with retrospective data. Only 26% of the identified articles were randomized controlled trials on the implementation of AI in clinical practice [1].

The aim of this review was to identify all current applications of AI in hand surgery and rehabilitation and elucidate their potential value in daily hospital routine. Although the term is nowadays often used as a synonym for machine learning, we did not want to limit this review to applications of machine learning. Throughout the literature search we encountered difficulties deciding which articles were relevant for this review. In order of covering a broad spectrum of applications which might be interesting to hand surgeons and therapists, we chose rather general search keywords. The search was conducted according to the Preferred Reporting Items for Systematic Reviews and Meta-Analysis Extension for Scoping Reviews (PRISMA-ScR) [6]. Since the inclusion/exclusion criteria did not provide clear thematic boundaries and the publications showed wide heterogeneity in content and quality the initially intended systematic review was found to be not feasible. In accordance with the aim of this study, to give the reader a meaningful overview of the topic, we conducted this scoping review with clustering of the articles into four groups of thematically related publications. This review being a “scoping review” and not a “systematic review” has to be considered a limitation of the study. Since the thematic boundaries and study characteristics [intended task of the artificial intelligence and performance metrics (AUC, dataset size, sensitiviy, specifity)] were most clear for the subgroup “Fracture detection using machine learning”, a detailed listing of these publications was provided (Table 3). With the other subgroups having rather blurry boundaries in terms of association to “artificial intelligence” and “hand surgery and rehabilitation”, we decided to elucidate these categories in a narrative way with citation of exemplary publications in order to achieve the objective of this review. This selection process not being strictly “systematic” is another limitation of this study.

Furthermore, this field is so rapidly evolving (exponential growth of publications on artificial intelligence in healthcare) that while a review is being conducted, written and revised, it is in danger of already being partially outdated/incomplete by the time of its publication. We consider this rather a characteristic of this research field than a limitation of the methodology of the review process.

Our conclusions on the current significance of AI in hand surgery:

Similar to orthopaedic surgery, the most established application of artificial intelligence in hand surgery is the creation of an *algorithm for automated detection and location of fractures* (e.g. distal radius fractures) *using machine learning.* This method is feasible for displaced fractures and at the time of this review (July 2021) the performance is mostly similar to the one of senior physicians and better than the one of unexperienced doctors. Yet, these algorithms often show difficulties in identifying undisplaced fractures (e.g. undisplaced scaphoid fractures). Judging on the recent evolution in this field we expect the performance of these algorithms to improve fast. Although the training of artificial intelligence takes much less time than the training of doctors, the performance is not yet good enough to replace humans. The limiting factors are mostly training datasets that are too small and contain heterogenous image data. We see the current value of this technology in prescreening patient data, helping clinicians in workflow prioritization (e.g. identifying highly displaced fractures with potential nerve compression or lesion and alerting the attending emergency room physician) or double checking the diagnosis in the background. Since these algorithms are usually only designed to detect (and at best locate) an injury, the potential help in “clinical decision-making” has been very limited up to now. Most articles highlight the feasibility and accuracy of artificial neural networks for a certain purpose, but lack to prove its practical usability. To change this, a broadly based project with a clear goal and vision of implementing AI in daily routine is necessary. Such a project should involve a large dataset of standardized (high quality, correctly oriented DICOM data) and labelled radiographic images, ideally with additional demographic data, accurate information about the injury, clinical symptoms, information about dexterity and activity level of the patient and the resulting treatment. Since the preparation and ground truth labelling of such datasets takes very much time, it has been the bottleneck of implementation in daily routine until now. Another difficulty is the connection between proficient IT knowledge and in-depth-hand surgery knowledge to develop such a project. To overcome this, close collaboration between data scientists and medical clinicians is required.

In contrast, in the field of *automated analysis of anatomical structures*, some currently available computer programs are intuitive and ready to use in daily routine. We utilize the analysis Bonelogic® 2 software (Disior Ltd, Helsinki, Finland) for automated segmentation of computed tomography data of distal radius fractures and automated analysis of intercarpal relationships (e.g. scapholunate distance). We see the benefit of this kind of software in supporting hand surgeons throughout the clinical workflow as the “surgeon’s third eye” and in preparation of treatment of complex cases (e.g. correction osteotomies), education of young surgeons and research purposes. Especially for less experienced surgeons, these programs can support better understanding of injuries and result in better treatment. With some further development and refinement of the automated segmentation software, these programs could not only detect the degree of displacement of fractures, but also their pattern and identify “key fragments” according to the osteoligamentous units theory. By combining this with information on the operative or non-operative treatment of former patients and adding it into a machine learning algorithm, a very powerful tool, which would add real, measurable value to the hand surgeon’s and emergency departments daily routine, could result. In our opinion, the implementation of these kinds of software solutions in clinical practice should be supported and funded by hospitals to ensure a good treatment quality.

The *other above-mentioned applications of artificial intelligence in hand surgery* (e.g. diagnosis of osteoporosis from hand radiographs using CNNs) are promising, but mostly in the context of experimental studies and therefore far from implementation in daily routine.

## Data Availability

Readers can access any data underlying the findings of this research article by contacting the authors.
